# Extracellular peptidase hunting for improvement of protein production in plant cells and roots

**DOI:** 10.3389/fpls.2015.00037

**Published:** 2015-02-06

**Authors:** Jérôme Lallemand, Frédéric Bouché, Carole Desiron, Jennifer Stautemas, Frédéric de Lemos Esteves, Claire Périlleux, Pierre Tocquin

**Affiliations:** ^1^Laboratory of Plant Physiology, Department of Life Sciences, University of LiègeLiège, Belgium; ^2^PhytoSYSTEMS, University of LiègeLiège, Belgium

**Keywords:** molecular pharming, peptidases, *Arabidopsis thaliana*, *Nicotiana tabacum*, root-secretion, suspension cells, *in silico* analysis

## Abstract

Plant-based recombinant protein production systems have gained an extensive interest over the past few years, because of their reduced cost and relative safety. Although the first products are now reaching the market, progress are still needed to improve plant hosts and strategies for biopharming. Targeting recombinant proteins toward the extracellular space offers several advantages in terms of protein folding and purification, but degradation events are observed, due to endogenous peptidases. This paper focuses on the analysis of extracellular proteolytic activities in two production systems: cell cultures and root-secretion (rhizosecretion), in *Arabidopsis thaliana* and *Nicotiana tabacum*. Proteolytic activities of extracellular proteomes (secretomes) were evaluated *in vitro* against two substrate proteins: bovine serum albumin (BSA) and human serum immunoglobulins G (hIgGs). Both targets were found to be degraded by the secretomes, BSA being more prone to proteolysis than hIgGs. The analysis of the proteolysis pH-dependence showed that target degradation was mainly dependent upon the production system: rhizosecretomes contained more peptidase activity than extracellular medium of cell suspensions, whereas variations due to plant species were smaller. Using class-specific peptidase inhibitors, serine, and metallopeptidases were found to be responsible for degradation of both substrates. An in-depth *in silico* analysis of genomic and transcriptomic data from *Arabidopsis* was then performed and led to the identification of a limited number of serine and metallo-peptidases that are consistently expressed in both production systems. These peptidases should be prime candidates for further improvement of plant hosts by targeted silencing.

## INTRODUCTION

Since 25 years and the demonstration by Hiatt et al. (1989) that the plant secretory pathway was able to carry out the folding and the assembling of complex eukaryotic proteins such as antibodies, plants have emerged as potential alternative hosts for the production of biopharmaceuticals. The amazing versatility of plant-based systems that have been developed (about a 100 platforms Schillberg et al., 2013), together with the economic and safety advantages they offer, aroused great expectations for this technology known as “molecular pharming.” However, it is only recently (2012) that the first plant-produced biopharmaceutical, a glucocerebrosidase produced in carrot cells as a treatment for the Gaucher’s disease (Shaaltiel et al., 2007), has been approved by the US Food and Drug Administration. Several reasons explain this slow industrial and market uptake: the relatively low and variable yields compared to the gold standard Chinese hamster ovary (CHO) cells for the production of complex human proteins (Twyman et al., 2013), the negative perception and restrictions on genetically modified organisms (GMOs; Schillberg et al., 2013), and the absence of a comprehensive regulatory framework (Fischer et al., 2013). High yields and regulatory compliance are key prerequisites to transform molecular pharming into an industrial success. Thus, while technologies were initially designed for transgenic plants grown in open fields, recent researches are rather focused on systems with a higher containment, which not only reduces the risk of GMOs release in the environment but also leads to a better control of the growing and production conditions (Paul and Ma, 2011; Schillberg et al., 2013).

In this context, systems based on plant cell- or tissue-cultures have emerged. They are either cell suspension cultures, mainly but not limited to tobacco Bright Yellow-2 cells (BY-2), or hairy root cultures induced by *Agrobacterium rhizogenes* (Schillberg et al., 2013). Both strategies share the advantage of producing biomass faster than whole plant cultures. Moreover, the product is often secreted into the culture medium, making its recovery easier and cheaper than extraction from the biomass (Twyman et al., 2013). Somehow intermediate between suspension and whole plant cultures, ‘floating’ systems based on the use of whole organisms that are fully or partly in contact with a culture medium (micro algae, moss, or aquatic plants) also have the advantages of being fully contained and allowing the secretion-based recovery of the product (Cox et al., 2006; Decker et al., 2014; Mathieu-Rivet et al., 2014). It is also the case of the rhizosecretion strategy where roots of hydroponically growing plants produce and secrete the recombinant protein into the nutrient solution. Such a system was initially proposed by Borisjuk et al. (1999) and later developed by Ma and colleagues (Drake et al., 2002, 2003, 2009).

One major limitation of secretion-based systems comes from proteolytic events frequently observed on the products (Pillay et al., 2014), a problem that is well documented for antibody production (e.g., Sharp and Doran, 2001; Drake et al., 2003; Niemer et al., 2014). Extracellular peptidases were demonstrated to be responsible for these degradations in various production systems: cell suspensions (Sharp and Doran, 2001), leaves (Hehle et al., 2011), or (hairy-)roots (Sharp and Doran, 2001; Drake et al., 2009). The extent of degradation depends on organs or production systems (Drake et al., 2009), developmental stages (De Muynck et al., 2009), culture medium (Häkkinen et al., 2014), or plant species (Magy et al., 2014). Cell wall proteome analyses, mainly performed in *Arabidopsis*, revealed that peptidases represent more than 10% of the extracellular proteins (Albenne et al., 2013). Moreover, Goulet et al. (2012) showed by a genomic analysis of *Arabidopsis*, rice, and *Nicotiana spp.* that, consistently across these species, a large proportion of peptidases are predicted to be targeted to the extracellular space. Considering that little is known about the function or the substrate of apoplastic peptidases (van der Hoorn, 2008; Tsiatsiani et al., 2012), their amount and diversity represent a major obstacle to the use of secretion in molecular pharming. However, counteracting strategies such as co-secretion of a single peptidase inhibitor (Komarnytsky et al., 2006; Robert et al., 2013) or the silencing of a single peptidase gene (Kim et al., 2008a; Mandal et al., 2014) have already proven efficient. To be successful, these strategies rely on a prior knowledge of the proteolytic activities likely to lead, in the operated production system, to the degradation of the target recombinant protein.

In the present paper, we aimed at cross-comparing the extracellular proteolytic activities of two production systems, cell culture and rhizosecretion, set-up from two species, *Arabidopsis thaliana* and *Nicotiana tabacum*. We specifically addressed this question in the case of human immunoglobulin (hIgGs) production. We hypothesized that *in silico* analyses of genomic and transcriptomic data obtained from a model species such as *Arabidopsis* could be merged with experimental results obtained from biochemical assays with existing production systems to provide robust insights about the major peptidases that limit hIgGs yields.

## MATERIALS AND METHODS

### PLANT CULTURES

Plants of *A. thaliana* cv. Columbia-0 (Col-0) and *N. tabacum* cv. Petit Havana SR1 were grown in hydroponics (Araponics, Belgium), at 20°C, with a relative humidity of 70%, a photoperiod of 10 h and a light intensity of 100 μmol m^-2^ s^-1^. Plant culture was adapted from Tocquin et al. (2003): seeds were sown on the top of seed-holders filled with a gel [0.5% Phytagel^TM^ (Sigma-Aldrich, St. Louis, MO, USA), 5 mM CaCl_2_, 0.15% polyvinylpyrrolidone (MW 10,000)] and the nutrient solution was prepared with Flora Series fertilizers (FloraBloom, FloraMicro and FloraGro; GHE, France), 0.5 mL per liter each. The solution was renewed after 5 weeks of cultivation.

### RHIZOSECRETOME HARVESTING

The direct analysis of the extracellular medium (EM) of hydroponically growing plants is hindered by the high dilution level of endogenous secreted proteins. Moreover, many extracellular proteins, including peptidases, are known to be weakly bound to the cell wall. In order to get a comprehensive overview of the peptidases that could be involved in the degradation of secreted recombinant proteins, we used a harvesting protocol adapted from cell wall proteomics studies (Boudart et al., 2005). 7 weeks after sowing, roots were briefly drained, harvested and weighed. The total fresh weight (FW) measured is comprised of the root biomass, the intercellular fluid and the adsorbed nutrient solution. In preliminary experiments, we estimated the drained FW after centrifugation (10 min, 2700 g) to be ~30 and ~80% of total FW for *A. thaliana* and *N. tabacum*, respectively. The water content was taken into account to calculate the volume of a NaCl stock solution that must be added to the fresh root to obtain a final concentration of 1 M. The root samples were then incubated for 1 h at 4°C in 1 M NaCl under strong agitation in order to recover extracellular compounds. Roots were thereafter centrifuged in a Pierce^TM^ Centrifuge Column (Thermo Scientific, Rockford, IL, USA) during 10 min at 2700 g and at 4°C. The collected samples, hereafter called rhizosecretomes, were either used freshly prepared or stored at –80°C until use.

### CELL CULTURES

Cells of *A. thaliana* cv. plant system biology-dark (PSB-D) were cultivated in a 250-mL Erlenmeyer flask containing 50 mL of a liquid medium [0.44% Linsmaier and Skoog Medium (Duchefa Biochemie, Haarlem, the Netherlands), 3% sucrose, 5.10^-5^% style="color:gray;" 1-naphthaleneacetic acid, 5.10^-6^% style="color:gray;" kinetin, pH 5.7 (KOH)] in the dark, at 25°C and under agitation at 90 rpm on a rotary shaker. Each week, fresh liquid medium was inoculated with a 10% inoculum of the former cell culture. Cells of *N. tabacum* cv. BY-2 were grown in the same conditions as *A. thaliana* cells, in an adapted culture medium [0.44% Murashige and Skoog salts (MP BIOMEDICALS, Solon, OH, USA), 3% sucrose, 0.02% KH_2_PO_4_, 2.5.10^-4^% style="color:gray;" thiamine, 5.10^-3^% style="color:gray;" myo-inositol, 2.10^-5^% 2,4-D, pH 5.8 (KOH)]. A 5% inoculum was used to inoculate fresh liquid medium each week. Both types of cells were kindly provided by Prof. Marc Boutry and his colleagues (Physiological Biochemistry Unit, Catholic University of Louvain, Louvain-la-Neuve, Belgium).

### EXTRACELLULAR MEDIUM HARVESTING

The EM of the 7-days-old *Arabidopsis* cell cultures was vacuum-filtered through a superposition of a glass fiber prefilter and a cellulose acetate filter, with a pore size of 0.2 μm (Sartorius Stedim Biotech GmbH, Goettingen, Germany); tobacco cell cultures were vacuum-filtered through three layers of Miracloth (Calbiochem; Merck KGaA, Darmstadt, Germany). The EM was either directly used or stored at –80°C.

### GELATIN ZYMOGRAPHY

Twenty micro liter of rhizosecretome or EM were incubated during 20 min at room temperature in a non-reducing loading buffer (1% SDS, 10% glycerol, 0.02% bromophenol blue, 60 mM Tris-HCl, pH 6.8, final concentrations) and loaded on a 10% polyacrylamide gel containing 0.05% gelatin. After an electrophoresis of 45 min at 180 V, the gel was washed 3 × 20 min in 2.5% Triton X-100 and incubated for 16 h in 10 mM MES, 0.1 mM ZnCl_2_, 5 mM CaCl_2_, 1% Triton X-100, pH 5.75 (KOH). The gel was then stained by colloidal Coomassie blue as described further.

### TARGET PROTEINS *IN VITRO* DEGRADATION

Five micro liter of rhizosecretome or EM were incubated during 6 h at 25°C in a buffer at a fixed value of pH [100 mM glycine (for pH values between 2 and 4.5) or 100 mM Tris (for pH values between 5 and 9), 100 mM MES, 0.1 mM ZnCl_2_, 5 mM CaCl_2_, 0.1 M dithiothreitol], with 1 μg bovine serum albumin (BSA; A7906, ≥98% purity, Sigma-Aldrich, St. Louis, MO, USA) or 1 μg immunoglobulin G from human serum (hIgGs; I4506, ≥95% purity, Sigma-Aldrich, St. Louis, MO, USA), in a final volume of 20 μL. Inactivation of peptidase classes was obtained by pre-incubating the rhizosecretome or EM in a buffer during 30 min at room temperature, with class-specific inhibitors: 5 mM PMSF (specific inhibitor of serine peptidases), 40 μM E-64 (specific inhibitor of cysteine peptidases), 16 μM Pepstatin A (specific inhibitor of aspartic peptidases) and 20 mM EDTA (specific inhibitor of metalloproteases). Mixes of all but one inhibitors were used, leaving only one peptidase class active per sample. 1 μg of BSA or human IgG was then added and incubated during 3 h (rhizosecretome) or 6 h (EM) at 25°C. After incubation, the target protein degradation profile was analyzed by SDS-PAGE and colloidal Coomassie blue staining.

### SDS-PAGE AND COLLOIDAL COOMASSIE BLUE STAINING

Protein samples were incubated during 10 min at 95°C in a reducing loading buffer (0.1 M dithiothreitol, 1% SDS, 10% glycerol, 0.02% bromophenol blue, 60 mM Tris-HCl, pH 6.8, final concentrations) before being separated by electrophoresis in a 10% polyacrylamide gel during 45 min at 180 V. The gel was then washed briefly in water, fixed during 1 h at room temperature in a fixation solution (30% ethanol, 10% acetic acid), washed 2 × 10 min in water, incubated overnight in a colloidal Coomassie blue staining solution [20% methanol, 8% ammonium sulfate, 1.6% phosphoric acid, 0.08% Coomassie Brilliant blue G-250 (Merck KGaA, Darmstadt, Germany)] and finally destained in a destaining solution (5% methanol, 7% acetic acid).

### *IN SILICO* ANALYSES

Fasta sequences of *Arabidopsis* peptidases were first retrieved from the FTP server of the MEROPS database ^1^. We then searched for corresponding sequences in the genome of *Arabidopsis* (TAIR10 ^2^) by BlastP. The best hit of each sequence was then further analyzed to remove non-perfectly matching sequences (>10% length difference, >2% mismatches).

*In silico* transcriptomic analyses were performed on *Arabidopsis* Affymetrix ATH1 raw data retrieved from the ArrayExpress database ^3^ using “roots” and “suspension cells” as queries. The resulting list of microarrays was manually sorted to remove experiments lacking comprehensive methodological informations. *The list of experiments included in the survey is available as a supplemental data file*. Within each experiment, we manually removed the microarrays performed on leaves and whole seedlings. The subsequent data analysis was performed using the R programming language (R Core Team, 2014). The “simpleaffy” Bioconductor package (Gentleman et al., 2004; Wilson and Miller, 2005) was used to read the raw data and perform the present/absent call on individual arrays using the detection.p.val() function. Genes were considered as being expressed when *p*-value <0.01. Within each experiment, we computed the proportion of arrays in which the gene of interest could be detected. Data were sorted using the list of peptidases defined above.

## RESULTS

### ACTIVE PEPTIDASES ARE SPECIES AND PRODUCTION SYSTEM SPECIFIC

Secreted peptidases of *Arabidopsis* (PSB-D cells and Col-0 strain) and tobacco (BY-2 cells and SR1 strain) were collected by filtration of 7-days-old cell suspensions (EM) or by centrifugation of hydroponic roots, harvested on 7-weeks old plants and incubated in a saline solution to solubilize apoplastic proteins [rhizosecretome (RZ)]. These harvesting timepoints were selected for maximum peptidase activity and diversity, based on preliminary time-lapse experiments (data not shown).

**FIGURE 1:**
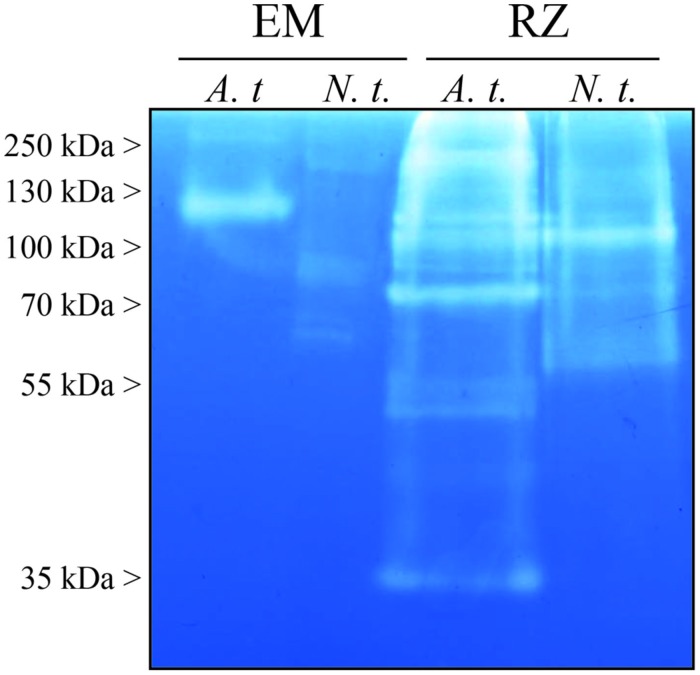
**Proteolytic activities analyzed by gelatin zymography of the extracellular media (EM) and the rhizosecretomes (RZs) of *Arabidopsis thaliana* (A.t.), and *Nicotiana tabacum* (N.t.).** Samples were collected from 7-days-old suspension cells (EM) or 7-weeks-old hydroponic cultures (RZ). 20 μl of EM or RZ were loaded on a SDS polyacrylamide gel containing 0.05% of gelatin. After electrophoresis, proteins were renaturated in the gel and the proteolytic activity was revealed after a 16-h incubation required for gelatin degradation. The presented result is representative of two independent experiments.

To get a first insight into the diversity of active peptidases, EM and RZ were analyzed by zymography with gelatin as substrate. As shown in **Figure 1**, the degradation patterns were highly different as regards with species and production systems.

The diversity of peptidase activities detected on zymogram was larger in RZ: more degradation bands were observed, over a wider range of molecular weights, than in EM (**Figure 1**). Moreover, in both RZ and EM setups, *Arabidopsis* samples displayed more degradation bands and bands with stronger activities than those from tobacco.

### pH-DEPENDENCE DEGRADATION OF SELECTED TARGETS MAINLY DIFFERS ACCORDING TO PRODUCTION SYSTEMS

**FIGURE 2:**
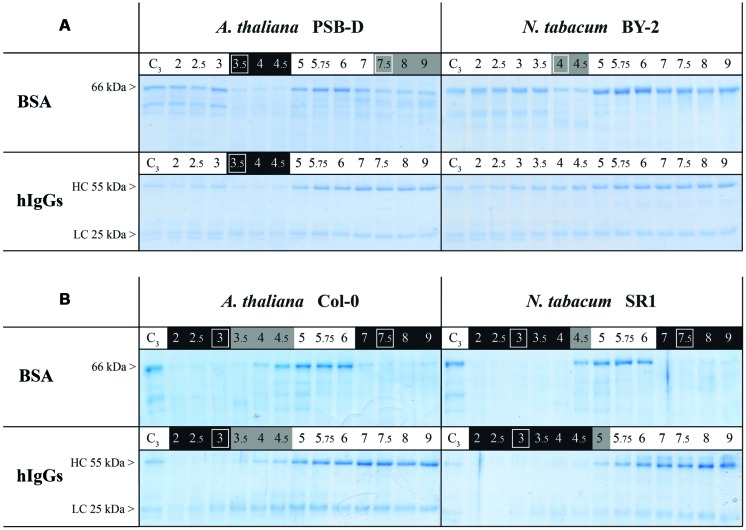
**Proteolytic activities in **(A)** extracellular medium of cell suspensions and **(B)** rhizosecretome in *A. thaliana* and *N. tabacum*.** Samples were collected from 7-days-old suspension cells (EM) or 7-weeks-old hydroponic cultures (RZ). 1 μg of BSA or hIgGs was incubated at 25°C during 6 h, in a buffer with a pH-value ranging from 2 to 9 and with 5 μl of secretome. It was then analyzed on reducing SDS-PAGE. C3 indicates control samples incubated at pH 3 after heat-inactivation of secretome proteins and thus shows maximum intensities of target-protein band(s). pH values highlighted in black or gray indicate pH ranges where, respectively, strong and moderate degradations were observed. The white frames indicate pH conditions selected for further analyses. The observed gradual decrease in hIgGs band intensities at acidic pH was not related to proteolytic events since this fade-out of the heavy chain band was also observed in control conditions. Each gel is representative of the results obtained from at least two independent experiments.

We then assayed *in vitro* the proteolytic activities of *Arabidopsis* and tobacco EM and RZ, against two target proteins: BSA and hIgGs, in pH conditions ranging from 2 to 9. We observed that the proteolytic activity was dependent upon the four experimental variables: the production system, the plant species, the target protein and the pH (**Figure 2**). However, the overall analysis of **Figure 2** clearly points out that the largest variation was due to the production systems, while species and target proteins only added limited variations to the overall pH-dependent activity. Obviously, the RZ production systems contained more proteolytic activity than cell suspensions EM (**Figure 1**).

The species- and target- effects were best observed in the cell suspensions systems (**Figure 2A**). In *Arabidopsis* PSB-D EM, degradation of BSA was mainly observed in acidic conditions (from 3.5 to ~5), but partial degradation was also observed at pH greater than 7. The other target, hIgGs, was also sensitive to *Arabidopsis* EM proteases at acidic pH of 3.5–4.5 but was less prone to degradation at higher pH, and remained intact in *Nicotiana* EM samples.

In RZ samples (**Figure 2B**), the same degradation profiles were obtained for both plant species but a strong target dependency was found: while BSA was degraded in acidic (2 to ~4) and neutral to basic (above 6) conditions, hIgGs were only degraded when incubated with RZ at pH below 5.

### SECRETED SERINE- AND METALLO-PEPTIDASES ARE ACTIVE AGAINST BSA AND hIgGs

In order to figure out which of the four main peptidase functional classes [aspartic (Asp), cysteine (Cys), metallo (Met), and serine (Ser) peptidases] were involved in the degradation of BSA and hIgGs in RZ and EM samples, we added specific peptidase inhibitors to the *in vitro* assays. Different mixtures of three specific peptidase inhibitors were used, in such a way that only one functional class was active in a single sample.

**FIGURE 3:**
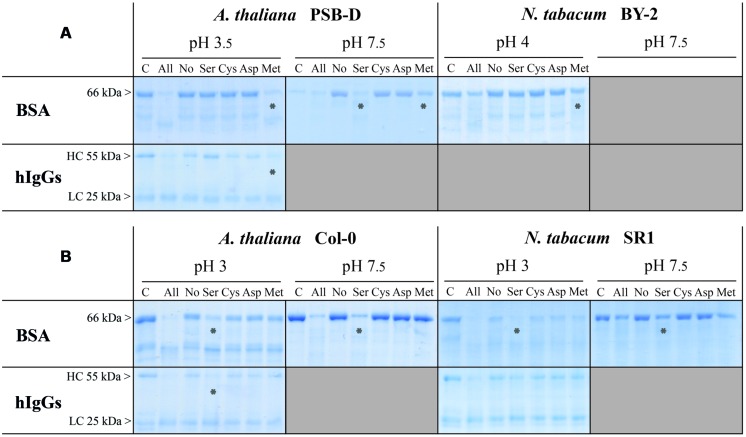
**Identification of peptidase classes responsible for target degradation in *A. thaliana* and *N. tabacum* EM or RZ.** Samples were collected from **(A)** 7-days-old suspension cells (EM) or **(B)** 7-weeks-old hydroponic cultures (RZ). Before a six- (EM) or a 3-h incubation (RZ) at 25°C with the target proteins (BSA or hIgGs), samples were pre-incubated for 30 min at 25°C without peptidase inhibitors (all proteases are active; ‘All’), or with a mix of four peptidase inhibitors (PMSF, E64, pepstatin A, and EDTA; none is active; ‘No’), or with a mix of three of these inhibitors. Omitting one of the inhibitors left only one peptidase class active: omitting PMSF, E64, pepstatin A, or EDTA left serine-(Ser), cysteine- (Cys), aspartic- (Asp), or metallo-(Met) peptidases active, respectively. The degradation profiles of the target proteins were observed after reducing SDS-PAGE. Lane labels refer to active peptidase classes. Lane C: the extracellular peptidases were heat-inactivated, without inhibitors, before the addition of the target protein. Stars indicate which protease classes degrade target proteins. Shaded areas show combinations that were not tested, because of the lack of target degradation (refer to **Figure 2**). Each gel is representative of the results obtained from at least two independent experiments.

As two major pH ranges were selected for degradation tests (acidic pH and neutral-basic pH, **Figure 2**), we performed these assays at pH 3 to 4 (depending on the system and species) and pH 7.5. In all assays, degradation of BSA and hIgGs was prevented by heat inactivation (**Figure 3**, ‘C’ lanes) or addition of the 4-inhibitor mix (**Figure 3**, ‘No’ lanes) indicating that any observed degradation was due to the peptidase activity of the samples. These treatments thus provided control samples showing maximum intensities of target bands whereas maximum degradation was observed in the absence of inhibitors (**Figure 3**, ‘All’ lanes).

In cell suspensions EM of both species, we found peptidase activities that were sensitive to PMSF and EDTA, respectively attributable to Ser and Met peptidases (**Figure 3A**). Met peptidases were the only observed active class at acidic pH, against both BSA and hIgGs, while Ser peptidases were mainly responsible for the proteolytic degradation occurring in neutral to basic pH in EM of *Arabidopsis* cells (**Figure 2**).

In RZ of *Arabidopsis* and *N. tabacum*, peptidases of the Ser family were responsible for BSA degradation observed at both acidic and neutral-to-basic pH conditions (**Figure 3B**). Because of the pH-dependent fade-out of the heavy chain band (see **Figure 2**), the degradation of hIgGs at acidic pH observed in **Figure 3** was more difficult to assign to a specific peptidase class but seemed to be, at least in *Arabidopsis*, also due to Ser peptidases.

### SERINE PEPTIDASES ARE PREDICTED TO BE THE MOST ABUNDANT CATALYTIC CLASS IN *Arabidopsis* SECRETOME

To go further toward the identification of putative Ser and Met peptidases that are responsible for degrading BSA and hIgGs *in vitro*, we performed an *in silico* analysis using genomic and transcriptomic data available for *Arabidopsis*.

**FIGURE 4:**
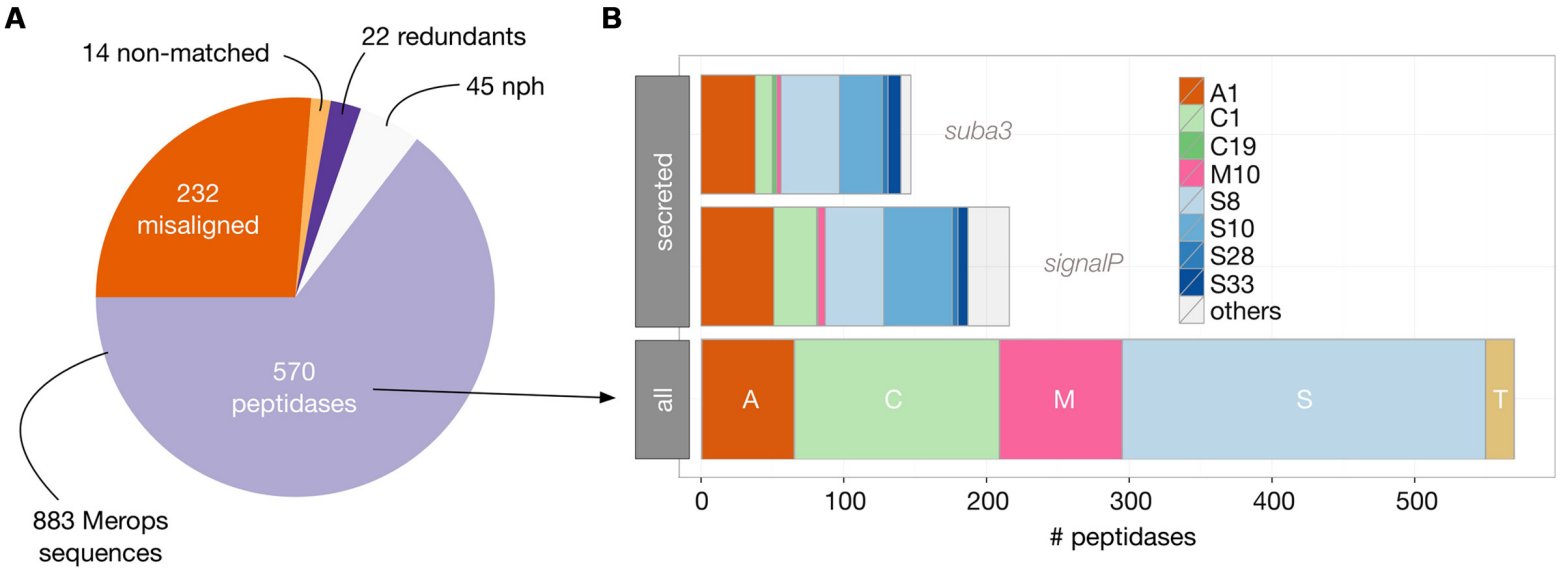
**Identification and distribution of *Arabidopsis* peptidases as inferred from MEROPS 9.11 database and TAIR10 genomic data. (A)** BlastP analysis (see Materials and Methods) of the 883 MEROPS sequences revealed 570 actual peptidases in *Arabidopsis* genome; the other MEROPS sequences did not have a perfect match in the genome (misaligned and non-matched), were duplicated (redundant), or were non-peptidase homologs (nph). **(B)** The 570 peptidases were classified according to their catalytic class (A, Asp peptidases; C, Cys peptidases; M, Met Peptidases; S, Ser peptidases; and T, Threonine peptidases) and family (from A1 to S33). Secreted peptidases were predicted either by the presence of a signal peptide (SignalP) or by their computed subcellular localization available in the SUBA3 database. Only families having at least two representatives are displayed as colored bars.

We first retrieved the 883 peptidase sequences available from the MEROPS 9.11 database (Rawlings et al., 2014) and filtered them for those corresponding to an existing locus in *Arabidopsis* genome [TAIR10, (Lamesch et al., 2012); **Figure 4**, see Materials and Methods].

This analysis revealed 570 unique loci corresponding to peptidases in *Arabidopsis* genome. Almost half of these peptidases belong to the Ser peptidase class (255). The Cys-, Met-, and Asp- classes follow with 144, 86, and 65 representatives, respectively. The subcellular localization of these peptidases was then evaluated to identify those that could be retrieved in the secretomes, i.e., in the extracellular space. A common strategy is to search for a N-terminal secretion signal in the sequence, using signalP or equivalent predictive tools (Petersen et al., 2011). With signalP 4.1 tool, 216 out of the 570 peptidases were predicted to be secreted. We next compared these results with those available from the Suba3 database, which is a curated subcellular location database for *Arabidopsis* proteins that combines information collected from literature, tagged protein experiments, protein–protein interaction datasets and results coming from 22 prediction programs (Tanz et al., 2013). As shown in **Figure 4**, a significant smaller amount of peptidases (147) were predicted to be extracellular by the Suba3 analysis compared with signalP 4.1, except for two families (S8 and S33) for which the opposite result was obtained. Among the 147 predicted secreted peptidases, 85 belong to the Ser-class and 6 to the Met-class and are thus putative candidates to explain proteolytic degradation detected in RZ and EM of *Arabidopsis*.

### MORE SECRETED PEPTIDASES ARE EXPRESSED IN HYDROPONICS vs. SUSPENSION CELLS

**FIGURE 5:**
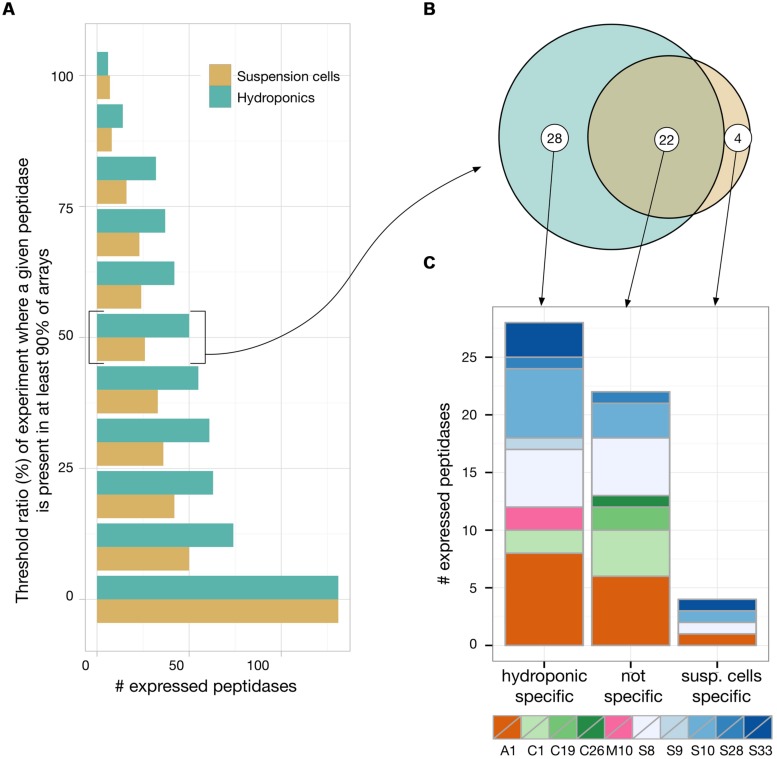
**Transcriptomic analysis of *Arabidopsis* secreted peptidases by a meta-analysis of publicly available microarrays data of hydroponic and suspension cell experiments.** A present/absent call (*p* < 0.01) was applied to each peptidase in each arrays of each experiment available. For each experiment, a peptidase was considered as ‘present’ if detected in at least 90% of its arrays. We then plotted in **(A)** the number of peptidases that were considered as present in at least a given proportion of the experiments. For peptidases that were found to be expressed in at least half of the experiments, we evaluated their specificity toward the expression context **(B)** and their distribution among the main represented peptidase families **(C).**

To evaluate which of those putative 147 secreted peptidases are most likely expressed in rhizosecretion and cell suspension production systems, we analyzed transcriptomic data available from the EBI Array express repository (Rustici et al., 2013). Experiments retrieved from a search with the ‘root’ and ‘suspension cells’ keywords were manually curated to select the most similar to ours, in terms of growing conditions and tissue sampling. Data gathered for hydroponics encompassed 16 experiments containing 310 arrays while we found 11 cell suspensions experiments with a total of 126 arrays. We then applied a ‘present/absent call’ function for each of the 147 peptidases, using the simpleaffy Bioconductor package (Wilson and Miller, 2005). A peptidase was considered as being expressed in an experiment if it was seen as ‘present’ in at least 90% of the arrays of this experiment (**Figure 5**). We chose this high cut-off to foster the selection of peptidases that were constitutively expressed, i.e., whose transcripts were present in almost all arrays of an experiment and thus independent of the experimental set-up. We then calculated, for both hydroponics and suspension cells, the proportion of experiments in which a peptidase was expressed. As shown in **Figure 5**, only about 50% of the putative secreted peptidases were found to be expressed in at least 10% of the experiments. We identified 50 peptidases expressed in a least half of the experiments in hydroponics and 26 in at least half of the experiments in suspension cells (**Figure 5**, threshold 50%). It is noteworthy that whatever the threshold, the number of expressed peptidases was always greater in hydroponics experiments than in suspensions cells.

With the threshold ratio set to 50% (i.e., expression detected in a least 50% of the experiments), only four peptidases were revealed as specifically expressed in suspension cells: one Asp and three Ser peptidases (**Figures 5B,C**). Two matrix metallopeptidases were found to be expressed in hydroponics but none in suspension cell cultures (AT1G59970, AT1G70170). With no threshold ratio set (i.e., expression detected in a least one experiment), two additional Met peptidases were detected in hydroponics (AT1G24140, AT1G71696). Two of these four Met peptidases were also expressed in suspension cells experiments (AT1G59970, AT1G71696). Ser peptidases were always the most represented catalytic class in transcriptomes (data not shown). At the 50% threshold, a total of 28 Ser peptidases were found to be expressed in hydroponics and/or suspension cells. Five of them were expressed in all experiments (threshold 100%) and are listed in **Table 1**; the complete list of secreted peptidases is available as Supplemental Data file.

**Table 1:** TAIR10 functional annotation of the five Ser peptidases that were found to be expressed in all hydroponics and/or suspension cells transcriptomic experiments analyzed in this study.

TAIR locus	MEROPS identifier	Peptidase family	Short description	Hydroponics	Suspension cells
AT1G17430	MER036056	S33	NA	16/16	5/11
AT1G71950	MER039047	S08	NA	16/16	11/11
AT2G05920	MER015427	S08	Subtilase family protein	16/16	11/11
AT4G12910	MER005597	S10	Serine carboxypeptidase-like 20	14/16	11/11
AT5G67360	MER001368	S08	Subtilisin-like serine protease (ARA12)	15/16	11/11

*The ‘Hydroponics’ and ‘Suspension cells’ columns indicate the number of experiments where the protease is expressed compared to the total number of transcriptomic experiments analyzed.*

## DISCUSSION

Unwanted degradation of recombinant proteins by endogenous peptidases is one of the major problems of plant-based heterologous production systems (Pillay et al., 2014). Peptidases are known to be involved in a multitude of processes from the cellular to whole organism level, but the exact functions and targets of most of them are still unknown (van der Hoorn, 2008). The strategies to prevent proteolysis thus generally rely on broad range inhibition of one or several catalytic classes, either by coexpression of peptidase inhibitors or direct gene silencing (Komarnytsky et al., 2006; Kim et al., 2008b; Goulet et al., 2010, 2012; Redkiewicz et al., 2012).

The increasing amount of ‘omics’ data provides an attractive starting point toward the identification of peptidases potentially at work in a given production system. However, this approach is rapidly limited by (1) the number of peptidases identified in genomes, (2) the limited knowledge about their functions, regulation and targets, and (3) the potential redundancy between peptidases of the same class, clan or family. Because peptidase activity is often controlled at the post-translational level, genomic and transcriptomic information are not sufficient. Moreover, functional redundancy hinders the identification of peptidases by simple activity-based assays. In this paper, we showed that meta-analyses of available genomic and transcriptomic data allow to reduce the huge list of peptidase candidates that results from activity-based assays, which essentially gives information about the catalytic class or the family.

We first focused on identifying active peptidase classes and addressing the question whether substantial differences exist between plant materials, production systems or target proteins, when assayed simultaneously. By using class-specific inhibitors, we showed that only Met and Ser peptidases were active against BSA and/or hIgGs, depending on the production system (RZ or EM) and the target protein.

Extracellular Met peptidases count only a few members, most of them belonging to the matrix metalloproteinases (MMPs) family (Marino and Funk, 2012). In our *in silico* analysis, only two MMPs were detected in a majority of hydroponic experiments, one of them being observed only in a few suspension cells experiments, together with a Zn^2+^ carboxypeptidase. Despite the scarcity of Met peptidases in secretomes, our results clearly showed that Met activity was present in EM of both *Arabidopsis* and BY-2 suspension cells, in agreement with previous reports for BY-2 (Delannoy et al., 2008; Schiermeyer et al., 2009; Mandal et al., 2010). The production system thus appears to be a strong determinant of proteolytic activities. The similarity between PSB-D and BY-2 cells overrides their species and tissue origins, which are *Arabidopsis* stem and tobacco root explants, respectively. By contrast, the two tobacco-based systems are very different in terms of pH dependence and active peptidases, despite their common species and tissue origins.

In contrast with Met peptidases, Ser peptidases are the most abundant class in plant cells and were therefore primarily targeted for plant-based production system improvement. Co-expression of Ser inhibitor, either as a second transgene or a fusion protein, improved, yet not totally, stability of recombinant proteins (Komarnytsky et al., 2006; Kim et al., 2008b; Goulet et al., 2010, 2012; Redkiewicz et al., 2012). Prior assessment of active peptidases is rarely performed and, if any, is usually done with non-specific targets (Rivard et al., 2006; Goulet et al., 2012). However, even closely related proteins, for example recombinant immunoglobulins, exhibit variable sensitivity to peptidases (Magy et al., 2014; Niemer et al., 2014), and hence end-product evaluation of peptidase activities is needed. The actual risk of proteolytic degradation is even more difficult to predict if several production systems or hosts are available, since each of them has its own peptidase assortment. This diversity was clearly illustrated here by comparing suspension cells and rhizosecretion in *Arabidopsis* and tobacco and was reported before (Magy et al., 2014; Pillay et al., 2014).

Merging activity assays with geno-transcriptomic data allowed us to narrow down the list of Ser peptidases potentially responsible for target degradations in *Arabidopsis* RZ and EM: out of 255 Ser peptidases identified in the genome, 85 were predicted to be extracellular and 25 were expressed in conditions similar to the production systems. Five of them were consistently expressed in suspension cells or hydroponics experiments included in the meta-analysis (**Table 1**), among which the serine carboxypeptidase SCP20 and the subtilisin-like ARA12 (**Table 1**). SCP20 was detected in the extracellular space of seedlings and leaves, and was reported to be strongly induced following fungal infection (Charmont et al., 2005; Floerl et al., 2012). ARA12 was frequently identified in cell wall proteomics studies either in EM of suspension cell cultures (Hamilton et al., 2003; Shen et al., 2013) or in seedlings and leaves (Boudart et al., 2005; Charmont et al., 2005; Fraser et al., 2005; Floerl et al., 2012). If the exact function of ARA12 remains unknown, its purification and biochemical characterization showed that it is a heat-stable peptidase functioning in a wide range of pH, from 3 to 7 with an optimum around 5 (Hamilton et al., 2003). These properties fit very well with the conditions in which we performed the activity assays presented here. Interestingly, a close *N. tabacum* homolog of ARA12 was identified in BY-2 EM (Navarre et al., 2012), indicating that production systems are similar, whatever the plant species, as also inferred from our cross-comparison.

Our cross-comparison of production systems and plant hosts, together with our *in silico* analysis of peptidases, consistently show that suspension cell cultures provide a less proteolytic environment for the production of recombinant proteins, especially antibodies. Even if less degradation was observed in BY-2 tobacco cells compared with *Arabidopsis* PSB-D cells, the relatively weak influence of the plant species permits to use model species such as *Arabidopsis* as a starting point for optimization of other host species. The top listed peptidases identified by our combined biochemical/bioinformatic analysis are thus prime candidates for new technology development, e.g., amiRNA- or MIGS multigene silencing, or nuclease assisted genome engineering (Schwab et al., 2006; Felippes et al., 2012; Voytas, 2013). These methods should soon be applicable to various plant species as the acquisition of genomic information increases quickly. Nevertheless, *Arabidopsis* is a competitive production system, even when compared with the well established *N. tabacum* BY-2 cells (Magy et al., 2014), and may even take a stronger position if one takes advantage of its technological advance for genetic engineering.

## Conflict of Interest Statement

The authors declare that the research was conducted in the absence of any commercial or financial relationships that could be construed as a potential conflict of interest.

## Footnotes

1 ftp://ftp.sanger.ac.uk/pub/MEROPS/current_release/protease.lib

2 ftp://ftp.arabidopsis.org/Sequences/blast_datasets/TAIR10_blastsets/TAIR10_pep_20110103_representative_gene_model_updated

3 http://www.ebi.ac.uk/arrayexpress/
